# *Cryptosporidium* Contamination and Attributed Risks in Yunlong Lake in Xuzhou, China

**DOI:** 10.1155/2017/4819594

**Published:** 2017-03-12

**Authors:** Yadong Kong, Ping Lu, Tao Yuan, Jinghui Niu, Zhaoji Li, Baisong Yang

**Affiliations:** ^1^School of Environment Science and Spatial Informatics, China University of Mining and Technology, Xuzhou 221000, China; ^2^Department of Architecture Equipment and Civil Engineering, Jiangsu Vocational Institute of Architectural Technology, Xuzhou 221116, China; ^3^JiangSu Collaborative Innovation Center for Building Energy Saving and Construct Technology, Xuzhou 221116, China

## Abstract

Swimming in surface water bodies (e.g., lakes, rivers) can expose the human body to substantial risk of infection by* Cryptosporidium*. These findings are from a one-year investigation on the occurrence and distribution of the protozoan parasite* Cryptosporidium* in Yunlong Lake, Xuzhou, China.* Cryptosporidium* oocysts were detected by immunofluorescence microscopy. From January to November of 2015, 180 samples (120 water samples and 60 sediment samples) were collected and analyzed. Among them, 42 (35%) water samples and 28 (47%) sediment samples tested positive for* Cryptosporidium*. The concentration of* Cryptosporidium* oocysts in the water samples was 0–8/10 L and 0–260/g in sediment samples. Results revealed that July was the highest risk period for both swimming and diving with an estimated probability of infection from swimming of greater than 18 per 10,000 swim sessions. It was concluded that swimming or diving in Yunlong Lake has a higher risk of* Cryptosporidium* infection than the acceptable risk level set by the United States Environmental Protection Agency. Thus, regular monitoring of water quality in recreation water bodies is strongly recommended.

## 1. Introduction

Diarrhea is the third leading cause of deaths in the developing countries. Each year, about 1.8 million people die from diarrheal diseases worldwide, and 90% of the victims are children under the age of five [1, 2].

Cryptosporidiosis is a type of the diarrheal illness caused by the infection of* Cryptosporidium*.* Cryptosporidium* is especially dangerous for infants, children, older people, pregnant women, and individuals with Acquired Immune Deficiency Syndrome. There has been an increased prevalence of cryptosporidiosis in densely populated areas in China, such as Beijing, Shanghai, Jiangsu, and Anhui Province [3, 4]. Most infections of* Cryptosporidium* stem from recreational water bodies [5]. Considering the risk of different ways of* Cryptosporidium* infection (i.e., drinking unboiled tap water and swimming and diving in the water bodies), swimming poses the highest risk of infection [6]. However, this exposure pathway for* Cryptosporidium* infection has received little attention in China.

Recreational water bodies can be contaminated by animal excretions, runoff from* Cryptosporidium*-contaminated soil, and discharge from wastewater treatment facilities and sewage systems [7]. A single infected animal excretion could contain billions of oocysts [5]. These oocysts are very resistant to many environmental stresses and can survive for a long period of time. Due to their small size and low settling velocity, they are very slow to settle out of watercourses [8]. For the above-mentioned reasons, swimmers health is highly jeopardized when they swim in recreational water bodies.

The primary purpose of this paper is to show the distribution of* Cryptosporidium* in the Yunlong Lake and to discuss the potential health risks while swimming or diving in this lake.

## 2. Methods

**Figure 1:**
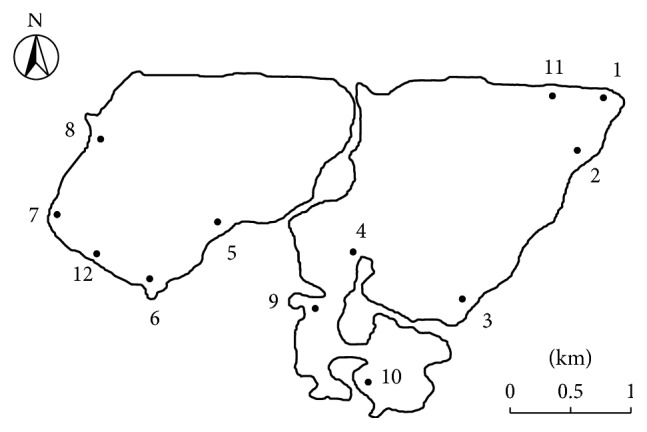
Sample collection sites.

Yunlong Lake is located in the southwest of Xuzhou City and has a surface area of 6.8 km^2^ and perimeter of 12 km. It is a recreational aquatic venue used for swimming and boating. A total of 120 water samples and 60 sediments samples were collected from 12 sampling sites along the lake (as shown in Figure 1). The sampling sites included restaurants, docks, fishing areas, boating training bases, and areas around living quarters. In each sampling site, duplicate water samples were collected from 0.1–2 meters below the water surface. Approximately 10 L of water was collected in a 10 L sterile plastic tank. Sediment samples were taken by a piston cylindrical sampler (KHT0204, Yinhua, Shangyu). Water and sediment samples were collected five times in January, March, July, August, and November 2015. The months for sampling, July and August, are the highest flow months of the year as well as the peak summer vacation months in China. Therefore, the proportion of people swimming in this two-month period is very high in comparison to the rest of the year. January and November were chosen because they are the driest months reported in this region.

Water samples were filtered through membrane filters with a pore size of 2 *μ*m by a vacuum device and concentrated using two centrifugation steps at 4,000 ×g at 4°C for 15 minutes, followed by two microcentrifugation steps at 15,000 ×g at 4°C for 5 minutes. The entire resuspended pellet was transferred into the slide wells. Sediment samples (20 g) taken from each site were mixed with distilled water and concentrated using two centrifugation steps at 2,000 ×g at 4°C for 20 min, discarding the supernatant and mixing the pellet with distilled water. The mixed solution was taken and transferred into a microcentrifuge tube and centrifuged at 2000 ×g at 4°C for 10 min [9].* Cryptosporidium *oocysts were numerated using an epifluorescence microscope under 200x magnification (Environmental Protection Agency (EPA) Method 1622) [10].

Exposure assessments consist of identifying the pathways (i.e., exposure routes: swimming and diving in the lake) and numbers of* Cryptosporidium* oocysts reaching a person and potentially leading to infection [6]. It has been reported about 18–37 mL of water is swallowed by swimmers per swimming event [11]. Additionally, 10.5–21.6% of people swim twenty times per year, and people swim an average of once a week during the summer [12]. Further, approximately 2.8–13 mL of water is swallowed for each dive and the estimated risk of diving was 2.1% for divers [13]. The studied area, Xuzhou, has a similar situation when compared with the previous research areas [6]. Therefore, the exposure frequency and population proportion were adopted for risk assessment in this study.

The exponential dose response model was used to estimate the infection risk of a single exposure [6], and the model is shown below:

(1) $$\begin{array}{c} P_{i}=1-e^{-rN}, \end{array}$$

where “*P*_*i*_” is the probability of infection and “*r*” shows the probability that* Cryptosporidium* can reach and infect a person. According to United States Environmental Protection Agency (USEPA), *r*_*Cryptosporidium*_ = 0.09 [14]. *N* is the dose, calculated by

(2) $$\begin{array}{c} N=\frac{CV}{rec}, \end{array}$$

where “*C*” is the detected* Cryptosporidium* concentration in water samples, with unit of number of* Cryptosporidium* per liter (n/L), rec is the recovery rate (rec was 0.3), and “*V*” is individual consumption of water in liters (L) [6, 15, 16].

An individual annual risk is defined as the risk associated with an individual's exposure to* Cryptosporidium* over a period of the year. The calculation of annual risk of infection, “*P*_*iy*_,” is shown as below:

(3) $$\begin{array}{c} P_{iy}=1-{\left(\;1-P_{i}\;\right)}^{n}, \end{array}$$

where “*n*” is the annual exposure number.

Beta probability density distribution was used to model the probability of annual illness that a healthy consumer is infected by* Cryptosporidium* [17]. The annual illness risk is shown as follows:

(4) $$\begin{array}{c} P_{ily}=P_{iy}\times P_{il}, \end{array}$$

where “*P*_*ily*_” is probability of annual illness and “*P*_*il*_” is probability of illness given infection.

Table 1 gives the model parameters for risk assessment of* Cryptosporidium* in Yunlong Lake.

## 3. Results

**Figure 2:**
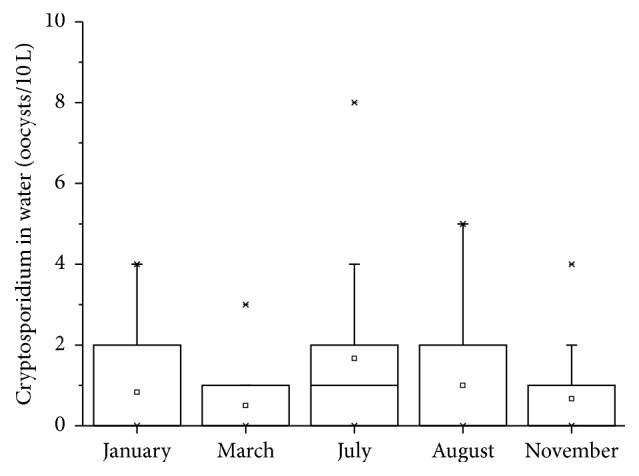
*Cryptosporidium* oocysts in water samples.

**Figure 3:**
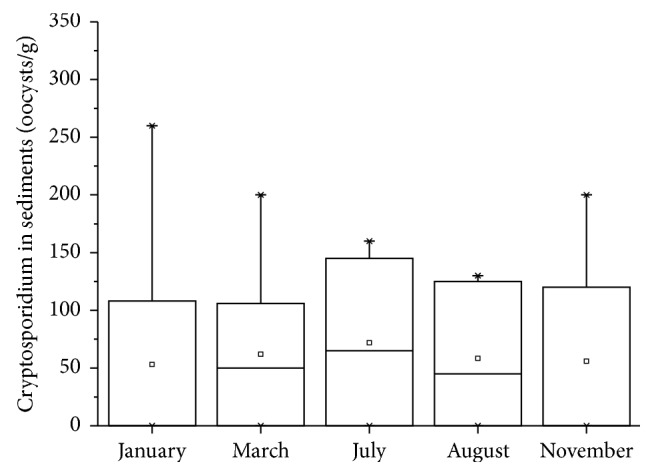
*Cryptosporidium* oocysts in sediment samples.

Figures 2 and 3 show the level of* Cryptosporidium* oocysts in water and sediment samples, respectively.* Cryptosporidium* oocysts were 0–8/10 L (with a mean value of 2.6/10 L) in water samples and 0–260/g (with a mean value of 129 oocysts/g) in sediment samples. The detection results of the samples are shown in Table 2. A higher incidence of* Cryptosporidium* positive samples was found in the rainy season (i.e., July-August) as compared to the results obtained in other periods. The seasonal pattern of* Cryptosporidium* in Yunlong Lake is similar to the Three Gorges Reservoir, China [6].* Cryptosporidium* in water samples of sampling sites 9 and 12 was detected positive for the whole year. It was also observed that water at sampling sites 9 and 12 was very cloudy.

Table 4 shows the risk of infection and illness by swimming and diving exposure in the lake according to the measured oocysts number per liter of the lake water. Swimming in July has the highest infection risk of 1.85 × 10^−3^ per time, while swimming in March has the lowest infection risk of 5.55 × 10^−4^ per time. Swimming in July has the highest yearly infection (3.64 × 10^−2^) and illness risks (1.82 × 10^−2^).

Table 5 shows the risk of infection and illness caused by diving exposure in the lake according to measured oocysts number per liter of the lake water. Diving in July has the highest infection risk of 6.51 × 10^−4^ per time, while diving in March has the lowest infection risk of 1.95 × 10^−4^ per time. Diving in July has the highest yearly infection (1.29 × 10^−2^) and illness risks (6.45 × 10^−3^).

## 4. Discussions

*Cryptosporidium* have been detected in source water bodies worldwide [15, 18–20]. However, cryptosporidiosis outbreak in recreational water is more frequent than in source water [5]. This study provides information on Yunlong Lake contamination by* Cryptosporidium*. The incidence of* Cryptosporidium* was not found to be higher than other water bodies reported in China (Table 3).* Cryptosporidium* was more frequently detected in sediments than that of surface water in Yunlong Lake (with positive rate of 47% and 35% for sediments and surface water, resp.) (Table 2). In the present study,* Cryptosporidium* concentration in sediment samples was not strongly correlated with those of water samples with a correlation coefficient of only 0.23. Oocysts cannot be homogeneously distributed throughout the water [21], which could help to understand the weak correlation between* Cryptosporidium* concentration in water and in sediments. An obvious seasonal contamination pattern was observed, with higher* Cryptosporidium* frequency in summer compared to the other seasons, which was similar to Xiao's report [6].

Modeling results indicated a potential health risk for people swimming or diving in Yunlong Lake. Compared to diving, swimming has the higher infection risk (Table 4). Similarly, An and coworkers reported that swimming is the primary risk of* Cryptosporidium* infection in rivers [22]. In regard to an acceptable risk level, an annual individual infection risk of 10^−4^ was suggested by the USEPA. The average infection risks of* Cryptosporidium* for swimmers and divers in Yunlong Lake were all greater than the USEPA's acceptable risk level (Tables 4 and 5).

A compilation of* Cryptosporidium* concentrations in water samples from other study areas is listed in Table 3. Average concentration of* Cryptosporidium* oocysts in the present study (2.6/10 L water) is comparable to the others. Watersheds from most of China have similar or higher* Cryptosporidium* positive rates in comparison with Yunlong Lake.

## 5. Conclusions 

On the basis of the above results and discussions, it was concluded that swimming in Yunlong Lake has a higher risk of* Cryptosporidium* infection than the acceptable risk level set by the USEPA. Multiple-site monitoring of recreational water quality and timely reporting of information regarding infection risk are strongly recommended based on the data showing that this lake is indeed contaminated by* Cryptosporidium*.

## Figures and Tables

**Table 1:** Model parameters for risk assessment of *Cryptosporidium.*

Variable description (unit)	Mean
January average oocysts concentration, sites 1–12 (per 100 L)	25
March average oocysts concentration, sites 1–12 (per 100 L)	15
July average oocysts concentration, sites 1–12 (per 100 L)	33
August average oocysts concentration, sites 1–12 (per 100 L)	30
November average oocysts concentration, sites 1–12 (per 100 L)	26
Water ingestion volume by swimming in the lake (mL/time)	37
Water ingestion volume by diving in the lake (mL/time)	13
Percentage of exposure swimmers (%)	16.05
Percentage of exposure divers (%)	2.1
Infectivity of annual exposures for swimming (times)	20
Infectivity of annual exposures for diving (times)	19.82
Probability of illness given infection	0.5

**Table 2:** Sample detection results for the 12 sites in Yunlong Lake, Xuzhou, 2015.

	Number of the positive samples detected	Positivity rate in percent
January		
Water	4	33.3%
Sediment	5	41.7%
March		
Water	4	33.3%
Sediment	6	50.0%
July		
Water	6	50.0%
Sediment	6	50.0%
August		
Water	4	33.3%
Sediment	6	50.0%
November		
Water	3	25.0%
Sediment	5	41.7%

**Table 3:** Occurrence of *Cryptosporidium* in water collected from various sites in China.

Location of sampling	Mean (oocysts/10 L)	Positive rate	Year of sampling	Reference
The Three Gorges Reservoir	1.92	100%	2011	[6]
River network system, Tongxiang	5.1	78.7%	2009	[23]
Water samples in Qinghai	N/A	25.4%	2011-2012	[24]
Waterworks in 33 major cities	0.7	N/A	2009–2011	[25]
Huangpu River	N/A	37.6%	2013-2014	[26]
Wastewater treatment plants in Harbin	N/A	31.3%	2009-2010	[27]
Source water, Shanghai	5.2	32%	2009-2010	[28]
Recreational water, Xuzhou	0.97	35%	2015	This manuscript

**Table 4:** Simulated risks by exposure events in Yunlong Lake (swimming).

Exposure routes	Probability of infection per time	Probability of infection per year	Probability of illness per year
Swimming in January	9.21 × 10^−4^	1.83 × 10^−2^	9.15 × 10^−3^
Swimming in March	5.55 × 10^−4^	1.10 × 10^−2^	5.51 × 10^−3^
Swimming in July	1.85 × 10^−3^	3.64 × 10^−2^	1.82 × 10^−2^
Swimming in August	1.11 × 10^−3^	2.20 × 10^−2^	1.10 × 10^−2^
Swimming in November	7.43 × 10^−4^	1.48 × 10^−2^	7.40 × 10^−3^

**Table 5:** Simulated risks by exposure events in Yunlong Lake (diving).

Exposure routes	Probability of infection per time	Probability of infection per year	Probability of illness per year
Diving in January	3.24 × 10^−4^	6.46 × 10^−3^	3.23 × 10^−3^
Diving in March	1.95 × 10^−4^	3.89 × 10^−3^	1.95 × 10^−3^
Diving in July	6.51 × 10^−4^	1.29 × 10^−2^	6.45 × 10^−3^
Diving in August	3.90 × 10^−4^	7.77 × 10^−3^	3.89 × 10^−3^
Diving in November	2.61 × 10^−4^	5.21 × 10^−3^	2.61 × 10^−3^

## Abbreviations

USEPA: United States Environmental Protection Agency.
